# Persistent upregulation of U6:SNORD44 small RNA ratio in the serum of breast cancer patients

**DOI:** 10.1186/bcr2943

**Published:** 2011-09-13

**Authors:** Hitesh N Appaiah, Chirayu P Goswami, Lida A Mina, Sunil Badve, George W Sledge, Yunlong Liu, Harikrishna Nakshatri

**Affiliations:** 1 Department of Surgery, Indiana University School of Medicine, West Walnut Street, Indianapolis, IN 46202, USA; 2 Department of Medicine, Indiana University School of Medicine, West Walnut Street, Indianapolis, IN 46202, USA; 3 Department of Pathology, Indiana University School of Medicine, West Walnut Street, Indianapolis, IN 46202, USA; 4 Department of Biochemistry and Molecular Biology, Indiana University School of Medicine, West Walnut Street, Indianapolis, IN 46202, USA

## Abstract

**Introduction:**

Serum microRNAs have the potential to be valuable biomarkers of cancer. This investigation addresses two issues that impact their utility: a) appropriate normalization controls and b) whether their altered levels persist in patients who are clinically free of the disease.

**Methods:**

Sera from 40 age-matched healthy women and 39 breast cancer patients without clinical disease at the time of serum collection were analyzed for microRNAs let-7f, miR-16, miR-21 and miR-155 using quantitative real-time PCR. U6 and 5S, which are transcribed by RNA polymerase III (RNAP-III) and the small nucleolar RNU44 (SNORD44), were also analyzed for normalization. Significant results from the initial study were verified using a second set of sera from 15 healthy patients, 15 breast cancer patients without clinical disease and 15 with metastatic disease, and a third set of 12 healthy and 18 patients with metastatic disease. U6 was further verified in the extended second cohort of 75 healthy and 68 breast cancer patients without clinical disease.

**Results:**

U6:SNORD44 ratio was consistently higher in breast cancer patients with or without active disease (fold change range 1.5-6.6, *p *value range 0.0003 to 0.05). This increase in U6:SNORD44 ratio was observed in the sera of both estrogen receptor-positive (ER+) and ER-negative breast cancer patients. MiR-16 and 5S, which are often used as normalization controls for microRNAs, showed remarkable experimental variability and thus are not ideal for normalization.

**Conclusions:**

Elevated serum U6 levels in breast cancer patients irrespective of disease activity at the time of serum collection suggest a new paradigm in cancer; persistent systemic changes during cancer progression, which result in elevated activity of RNAP-III and/or the stability/release pathways of U6 in non-cancer tissues. Additionally, these results highlight the need for developing standards for normalization between samples in microRNA-related studies for healthy versus cancer and for inter-laboratory reproducibility. Our studies rule out the utility of miR-16, U6 and 5S RNAs for this purpose.

## Introduction

MicroRNAs (miRNAs) are a class of multifunctional, small (18 to 25 nucleotides) non-coding RNA molecules [1,2]. To date, approximately 940 miRNAs have been described [3]. Their functions include epigenetic control of gene expression, mRNA degradation, and suppression of mRNA translation [4]. These diverse functions of miRNAs are necessary for normal development, metabolism, cellular differentiation, proliferation, cell cycle control, and cell death. Aberrant miRNA expression or activity or both have been implicated in a variety of human diseases, including cancer [5].

Several studies have analyzed miRNA expression patterns in primary tumors of various types, and specific subtypes of cancers could be easily differentiated on the basis of the expression pattern of these miRNAs [6]. Recent studies have identified miRNAs in extracellular space, mainly through ceramide-dependent secretory exosomes or microvesicles [7-9]. Additionally, secreted miRNAs have been shown to be in the Argonaute2 protein complex, which confers stability [10]. These secreted miRNAs are transported through high-density lipoprotein (HDL) and enter heterotypic cells to alter migration/invasive properties [7,8,11-13]. However, secretion or packaging of miRNAs into the exosomes is a selective process as the level of miRNA in exosomes secreted by a cell type does not always correlate with the intracellular levels of the corresponding miRNA [14]. Specific cellular proteins, most of which are RNA-binding proteins, are suggested to be involved in exosomal secretion of miRNAs and their stability in circulation [15].

Several reports describe differential blood/plasma/serum miRNA levels between healthy people and those with various diseases, including cancer [7-9,14,16-25]. Serum miRNA was first reported in diffuse large B-cell lymphoma; sera of patients contained higher levels of miR-155, miR-210, and miR-21 [25]. Elevated serum miR-21 levels correlated with good prognosis. Similar studies in prostate cancer revealed elevated levels of miR-141 in the plasma of patients with cancer compared with healthy subjects [24], although the same result was not obtained in another study [23]. A four-miRNA predictive profile from serum was described recently for non-small-cell lung cancer [22]. There are limited studies on breast cancer. One study reported higher serum levels of miR-155 in patients with progesterone receptor-positive (PR^+^) breast cancer compared with patients with PR^- ^breast cancer [26]. Two recent studies reported elevated levels of miR-195 and let-7a in the whole blood of patients with breast cancer; levels of these miRNAs declined after surgical removal of tumors, suggesting that they were tumor-derived [20,21]. Elevated levels of miR-195 in the whole blood appear to be unique to breast cancer [21]. Elevated levels of plasma miR-122 and miR-192 were reported after acetaminophen-induced liver injury, suggesting that tissues that are enriched for specific miRNAs may release them upon injury [27]. Patients with atherosclerosis display an HDL-associated miRNA profile that is distinct from that of healthy subjects [11].

It is postulated that the miRNAs are released into circulation either actively by the tumor cells or passively as a result of tumor cell death and lysis [28]. However, this does not explain low serum levels of some miRNAs in patients with cancer compared with healthy controls. For example, plasma of patients with acute myeloid leukemia shows low levels of miR-92a compared with healthy subjects despite high levels of this miRNA in leukemic cells [19]. In the sera of patients with lung cancer, 28 miRNAs are missing and 63 new miRNA species are detectable compared with healthy subjects [18]. Similarly, sera of patients with ovarian cancer show elevated levels of five miRNAs and decreased levels of three miRNAs compared with healthy subjects [17]. These observations raise questions of whether serum miRNAs in patients with cancer are directly derived from tumor cells or an indirect consequence of effects of cancer on other tissues, which then release miRNA into circulation. Given that the tumor often represents a very tiny portion of the body mass, microvesicles/exosomes secreted from the tumor cells are less likely to be sufficient enough to change the miRNA profile in a large volume of blood (5 L in a 72-kg person). Systemic effects of cancer on distant organs could easily result in a differential serum miRNA profile in patients with cancer. More importantly, these changes in serum profile could persist even after the patient is 'disease-free' if an epigenetic mechanism is involved in the systemic effects. In the latter situation, miRNAs would be poor markers of active disease.

To address these issues, we determined the levels of breast cancer-associated miRNAs in the sera of healthy subjects and breast cancer patients who were considered clinically cancer-free at the time of serum collection. Further validation of significant initial results was performed (a) with an independent sample set comprising serum from healthy subjects, clinically disease-free patients with breast cancer, and patients with overt metastasis and (b) with a set with serum from healthy subjects and patients with active metastasis. We report that SNORD44, a small nucleolar RNA (also called RNU44), is similar in the sera of healthy subjects and clinically cancer-free patients with breast cancer. However, levels of U6 (also called RNU6-1), which is commonly used for the purpose of normalization between samples, and U6/SNORD44 ratio were elevated in the sera of breast cancer patients who did not have active disease. Elevated U6 was detected in the sera of patients with estrogen receptor alpha-positive (ERα^+^) and of those with ER^- ^breast cancer. Sera of patients with overt metastasis also showed elevated U6 or U6/SNORD44 ratio when compared with healthy women. Taken together, these results suggest that elevated U6 serum levels represent persistent systemic effects of breast cancer attained during cancer progression.

## Materials and methods

### Sample processing and RNA extraction

All sera were obtained from Indiana University Simon Cancer Center's Komen Tissue Bank. Patients gave informed consent to participate in the study, and the Indiana University institutional review board that evaluates studies involving human subjects approved the study. All samples were collected in accordance with standard operating procedure, which is detailed in the tissue bank website [29]. More information on serum collection is provided as Additional file 1. RNA was isolated from 250 μL of serum by using the mirVana kit (Ambion, part of Applied Biosystems, Foster City, CA, USA) in accordance with the protocol of the manufacturer. RNA was eluted with 70 μL of RNase-free water, and a NanoDrop ND-1000 (NanoDrop, Wilmington, DE, USA) was used to measure the concentration of RNA. Although it was reported previously that serum miRNAs are stable and can withstand repeated freeze-thawing [24], consistent results were obtained only when samples from healthy subjects and patients with cancer were handled similarly.

### Quantitative reverse transcription-polymerase chain reaction

In the first series of experiments, 5 μL of RNA was reverse-transcribed to cDNA in a final volume of 15 μL by using a Taqman miRNA reverse transcription kit (Applied Biosystems). In the additional cohorts, 25 ng of RNA was used for reverse transcription. Quantitative polymerase chain reaction (qPCR) was performed by using Taqman universal PCR mix (Applied Biosystems) and specific primers on the qPCR instrument (Applied Biosystems). Primers for U6 (catalog number 001973), miR-16 (#000391), miR-21 (#000397), miR-155 (#000479), and miR-195 (#000494) were purchased from Applied Biosystems, whereas 5S primer (#201509) was purchased from Exiqon (Vedbaek, Denmark). SNORD44 primers (MPH01658A-200) were purchased from SABiosciences (Frederick, MD, USA). In some experiments, SNORD44 primers from Applied Biosystems (also called RNU44, #001094) were used. Each amplification reaction was performed in duplicate in a final volume of 20 μL containing 2 μL of the cDNA. qPCR of sera from healthy subjects and patients with cancer for a particular probe was in the same plate in all but extended cohort 2 to limit mechanical errors. The expression levels of miRNAs, U6, and 5S were normalized to SNORD44 or miR-16 and were calculated using the 2^-ΔΔ^Ct method.

### Statistical analysis

Expression levels of serum miRNAs were compared by using the Mann-Whitney U test. A *P *value of less than 0.05 was considered statistically significant.

## Results

### Patient characteristics

Table 1 provides details of patient characteristics, including age at cancer diagnosis, age at serum collection, tumor types, ER/PR status, treatments received, and lymph node positivity of the experimental set (cohort 1). Age range of healthy volunteers (all women) is also shown. All patients except one were clinically free of overt metastasis at the time of serum collection. Patient characteristics of two other validation cohorts are presented in Tables 2 and 3.

**Table 1:** Characteristics of healthy volunteers and patients (experimental cohort)

	Healthy subjects	All patients		ER/PR^+^		ER/PR^-^	
Number	40	39		24		12	3^a^
Age, years^b^		Diagnosis	Analysis	Diagnosis	Analysis	Diagnosis	Analysis
Mean		44	49.9	45.5	51.1	40.9	46.2
Median	20-70	43	50	46	53	40	45
Range		28-64	30-67	28-64	30-67	28-56	36-63
Node-positive/ Node-negative	N/A	14/23		8/16		5/7	
Tumor type	N/A						
Pre-invasive		9		5		2	
Invasive		28		17		10	
Treatment							
Radiation		24		13		8	
Chemotherapy		28		16		10	
Hormone		23		19		2	

^a^Estrogen receptor/progesterone receptor (ER/PR) status of remaining three patients is unknown. ^b^Ages at the time of initial diagnosis and at the time of serum collection for analysis are indicated. Patients in this cohort were clinically disease-free at the time of serum collection. N/A, not available.

**Table 2:** Characteristics of healthy volunteers and patients with breast cancer during diagnosis and experimental analysis (cohort 2)

	Healthy subjects	All patients		Non-metastatic		Metastatic	
Number	15	29	1^a^	15		14	1^a^
Age, years^b^		Diagnosis	Analysis	Diagnosis	Analysis	Diagnosis	Analysis
Mean	53.26	46	52.06	48.06	52.13	43.78	52
Median	53	45	51	48	51	42	53
Range	47-69	23-75	23-80	38-71	46-76	23-75	23-80
Treatment							
Radiation		18		8		10	
Chemotherapy		19		9		10	
Hormone		13		6		7	
Only ER^+^/PR^+ ^patients		Total		Non-metastatic		Metastatic	
Number		23		13		10	
Age, years		Diagnosis	Analysis	Diagnosis	Analysis	Diagnosis	Analysis
Mean		46.34	52.08	48.69	52.38	43.3	51.7
Median		47	51.0	48	51	42	51
Range		23-75	23-80	38-71	46-76	23-75	23-80

^a^Age and metastatic status of patient are unknown. ^b^Ages at the time of initial diagnosis and at the time of serum collection for analysis are indicated. ER, estrogen receptor; PR, progesterone receptor.

**Table 3:** Ages at cancer diagnosis and sample collection of patients with active disease (cohort 3)

	All patients with metastasis		Bone metastasis		Lung/Liver metastasis	
Number	18		8		7	
Age, years	Diagnosis	Analysis	Diagnosis	Analysis	Diagnosis	Analysis
Mean	43.7	54.9	42.0	54.3	45.2	55.5
Median	43	57.0	42	54.5	43	57
Range	35-58	35-78	35-49	35-78	35-58	45-62
Treatment						
Radiation	11		5		6	
Chemotherapy	9		4		5	
Hormone	8		3		5	

Metastasis in three patients was in distant organs other than lung, liver, and bone. Eleven patients had estrogen receptor-positive/progesterone receptor-positive tumors. Metastasis was more common in bone, lung, and liver.

### miRNA and small RNA expression analyses

**Figure 1:**
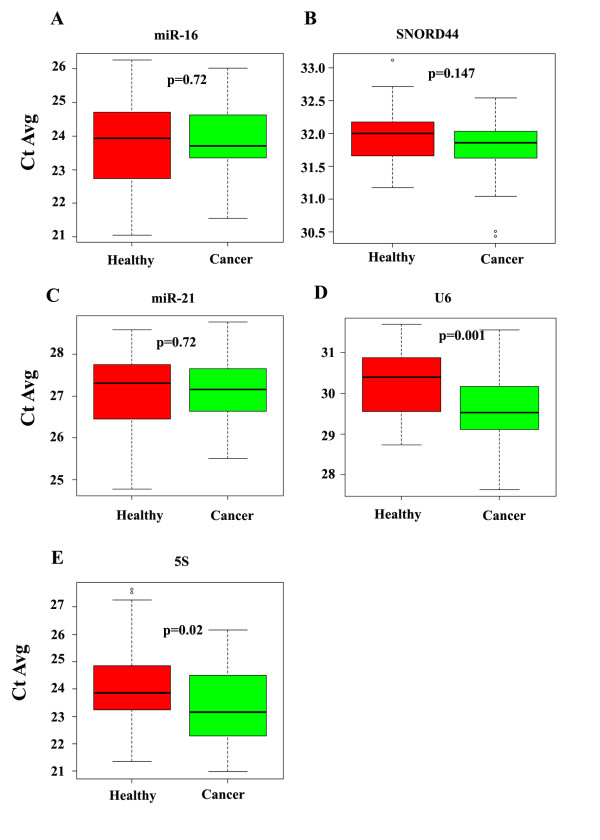
**Levels of miRNAs and small RNAs in sera of healthy volunteers and patients with breast cancer**. **(a) **MicroRNA 16 (miR-16) levels in the sera of healthy volunteers and patients with breast cancer. **(b) **Small nucleolar RNA 44 (SNORD44) levels in the sera of healthy volunteers and patients with breast cancer. **(c) **MiR-21 levels in the sera of healthy volunteers and patients with breast cancer. **(d) **U6 small RNA levels in the sera of healthy volunteers and patients with breast cancer. **(e) **5S small RNA levels in the sera of healthy volunteers and patients with breast cancer. Average cycle threshold (CT) values are shown; the lower the CT value, higher the expression. Patients in this cohort were clinically disease-free at the time of serum collection.

Earlier studies showing the presence of miR-21 and miR-155 in the serum/plasma of patients with cancer [25,26] prompted us to evaluate their levels in the sera of healthy subjects and patients with breast cancer. U6, 5S, miR-16, RNU66, RNU49, RNU19, and SNORD44 levels were also analyzed in these samples to identify a small RNA expressed at a similar level in equal volume of sera from both healthy subjects and patients with cancer to serve as a normalization control. Among these, miR-16 has previously been used as a normalization control for serum miRNA profiling studies [25]. RNU66, RNU48, and RNU19 were undetectable. miR-16 is one of the most abundant miRNAs in the serum (average cycle threshold (CT) of 24) compared with any other RNA analyzed, and the abundance of this miRNA in the serum was similar between healthy subjects and patients with cancer (Table 4 and Figure 1a). Although SNORD44 was present at lower levels than miR-16 (average CT of 32), its levels were similar in the serum of healthy subjects and patients with breast cancer (Table 4 and Figure 1b). Unlike in diffuse B-cell lymphoma [25], miR-21 did not show any differences between the two groups, although it is an abundant miRNA (average CT of 27) (Table 4 and Figure 1c). Let-7f and miR-155 were not considered for further analyses, because of higher CT values (> 30). The levels of U6 and 5S, in contrast to those of the above RNAs, were higher in the serum of patients with cancer compared with healthy subjects (Table 4 and Figure 1d, e). These results provided us the first indication of differential levels of circulating U6 and 5S in patients with cancer.

**Table 4:** Differences in the levels of U6, 5S, and other RNAs between healthy subjects and patients with cancer

Small RNA	*P *value	Fold change (cancer/healthy)
U6	0.001	2.42
5S	0.017	1.7
miR-21	0.72	-1.04
miR-16	0.72	-1.06
SNORD44	0.147	1.08
U6-miR-16	0.0047	2.58
U6-SNORD44	0.0028	2.22
5S-miR-16	0.02	1.82
5S-SNORD44	0.0599	1.53
miR-21-miR-16	0.93	1.01
miR-21-SNORD44	0.36	-1.14

In this experimental cohort, patients were cancer-free at the time of serum collection. miR-16, microRNA 16; miR-21, microRNA 21; SNORD44, small nucleolar RNA 44.

**Figure 2:**
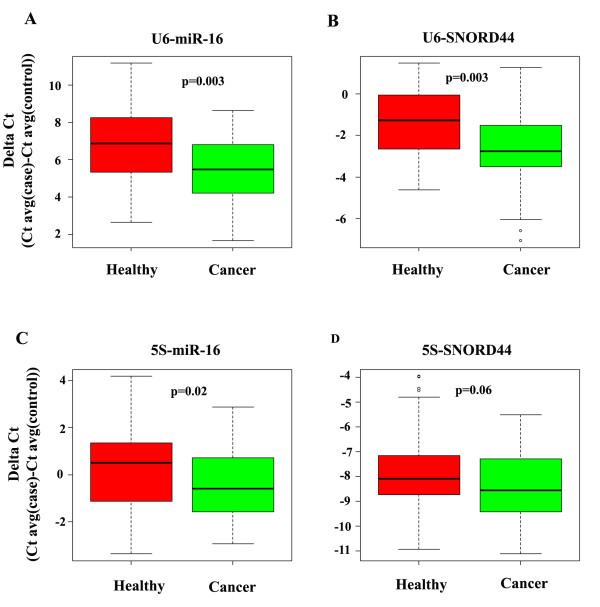
**U6 and 5S levels in the sera after normalization with microRNA 16 (miR-16) or small nucleolar RNA 44 (SNORD44)**. Delta cycle threshold (CT) method (CT average of U6 or 5S minus CT average of miR-16 or SNORD44) was used for this analysis; the lower the delta CT, the higher the expression. The statistical significance of differential expression and the fold change between groups are presented in Table 4.

Since miR-16 and SNORD44 levels were similar between two groups, we determined whether the differences in U6 and 5S levels between healthy subjects and patients with cancer retain statistical significance if miR-16 and SNORD44 are used as normalization controls. Indeed, the levels of U6 and 5S were significantly higher in the sera of patients with cancer compared with healthy subjects when miR-16 was used for normalization (Table 4 and Figure 2a-d). Differences in U6 levels remained significant when SNORD44 was used for normalization; however, differences in the levels of 5S did not reach significance under similar analysis (*P *= 0.06) (Table 4).

### Serum U6 and 5S in relation to ER/PR and nodal status of primary tumors

**Figure 3:**
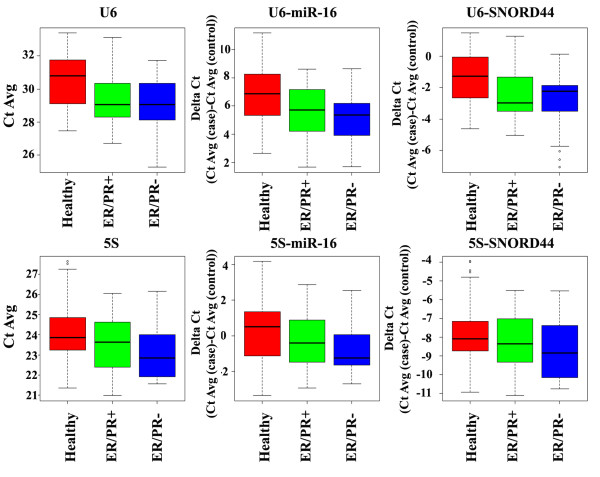
**Levels of U6 and 5S with or without normalization in the sera of patients who were diagnosed with estrogen receptor/progesterone receptor-positive (ER/PR^+^) or ER/PR^- ^tumors**. The statistical significance of differential expression and the fold change between groups are presented in Table 5.

We next determined whether the upregulation of U6 and 5S observed above is unique to specific subtypes of breast cancer. ER/PR status of 36 tumors, out of 39 patient samples used in the study, was known; 24 and 12 were ER/PR^+ ^and ER/PR^-^, respectively. In this subgroup analysis, U6, but not 5S, levels were higher in the sera of patients with ER/PR^+ ^breast cancer compared with healthy subjects with or without normalization with miR-16 and SNORD44 (Table 5 and Figure 3). Despite a small sample size, sera of patients with ER/PR^- ^demonstrated elevated U6 and 5S levels compared with healthy subjects when miR-16 or SNORD44 was used as an internal control (Table 5 and Figure 3). miR-21 level was similar between healthy subjects and patients with cancer of either subtype. Note that the differences in the levels of these RNAs between ER/PR^+ ^and ER/PR^- ^were not statistically significant (data not shown).

**Table 5:** Cancer subtype-specific differences in U6, 5S, and other RNAs compared with healthy subjects

Small RNA	ER^+^/PR^+ ^cancer versus healthy		ER^-^/PR^- ^cancer versus healthy	
	*P *value	Fold change	*P *value	Fold change
U6	0.01	2.16	0.004	3.03
5S	0.087	1.52	0.02	2.1
miR-21	0.52	-1.09	0.79	1.05
miR-16	0.614	-1.11	0.962	1.01
SNORD44	0.21	1.09	0.29	1.09
U6-miR-16	0.02	2.4	0.02	3.00
U6-SNORD44	0.02	2.0	0.008	2.77
5S-miR-16	0.07	1.7	0.05	2.08
5S-SNORD44	0.196	1.4	0.055	1.92
miR-21-miR-16	0.96	1.01	0.91	1.03
miR-21-SNORD44	0.273	-1.19	0.85	-1.04

Small RNA levels in the serum of patients with estrogen receptor/progesterone receptor-positive (ER/PR^+^) or ER/PR^- ^cancer were compared with those in the serum of healthy volunteers (experimental cohort). miR-16, microRNA 16; miR-21, microRNA 21; SNORD44, small nucleolar RNA 44.

Previous studies have shown that patients with lymph node-positive breast cancer have lower blood let-7a levels compared with patients with lymph node-negative breast cancer, suggesting the influence of metastasis in regulating serum miRNA levels [20]. In our study, comparison of U6 and 5S RNA between sera of node-positive (*n *= 14) and node-negative (*n *= 23) patients did not reveal any significant difference (data not shown). These results suggest that the elevated U6 level observed in the sera of cancer patients who were free of overt metastasis is less likely to be due to undetectable micrometastasis.

### U6 levels are elevated in sera of patients with breast cancer, irrespective of metastasis

**Figure 4:**
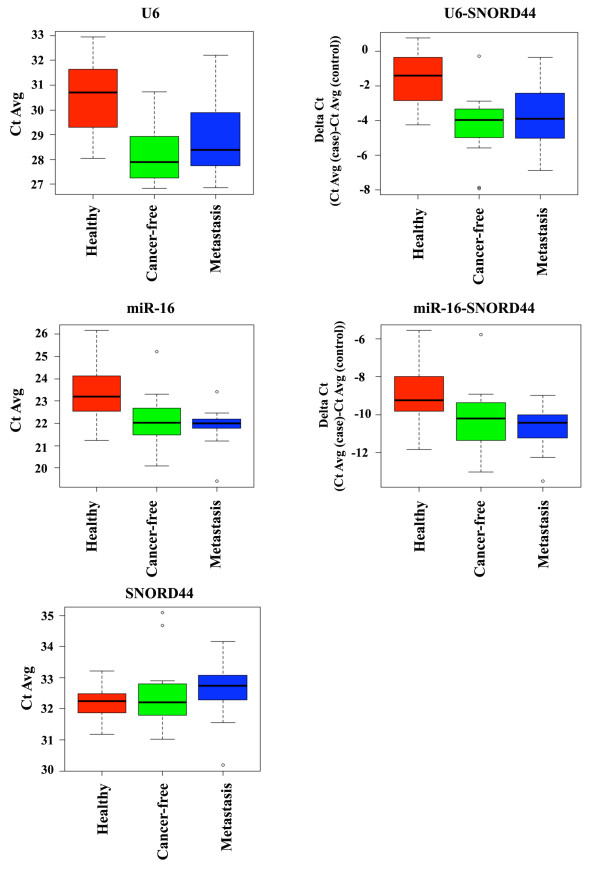
**U6, U6/small nucleolar RNA 44 (SNORD44) ratio, 5S, 5S/SNORD44, microRNA 16 (miR-16), miR-16/SNORD44 ratio, and SNORD44 levels in the serum of healthy subjects, patients who are clinically cancer-free, and patients with active metastasis**. Ages at initial diagnosis and serum collection are presented in Table 2. The statistical significance of differential expression and the fold change between groups are presented in Table 6.

We collected sera from 15 healthy subjects, 15 clinically disease-free patients with breast cancer, and 15 patients with stage IV metastatic disease. RNA from the three groups (cohort 2) was prepared at the same time from an equal volume of sera. The cDNA was prepared with 25 ng of RNA and analyzed for the levels of U6, 5S, miR-21, and miR-16. In this cohort, as with the first cohort, U6 level was significantly elevated in the sera of clinically disease-free patients with breast cancer without normalization (Table 6 and Figure 4) (5.35-fold, *P *= 3.09 × 10^-5^) or with normalization with SNORD44 (6.6-fold, *P *= 0.00028). In this experiment, unlike in the previous experiments, we found a statistically significant increase in miR-16 levels in the sera of disease-free patients with breast cancer compared with healthy subjects with or without normalization with SNORD44 (Figure 4). These results suggest that miR-16 is not appropriate for normalization. When the analysis was restricted to patients with metastasis versus healthy subjects, differences in U6 levels remained significant without normalization (Table 6 and Figure 4) (4.31-fold, *P *= 0.0005) and after normalization with SNORD44 (4.8-fold, *P *= 0.004). Note that differences in U6 levels between clinically disease-free patients with breast cancer and patients with metastasis were not significant. Additionally, SNORD44 levels were similar in all three groups (Table 6 and Figure 4).

**Table 6:** Comparison of expression levels of U6, 5S, and other RNAs in the serum

Column ID	*P *value	Fold change
Metastasis versus healthy		
U6	0.0005	4.31
U6-SNORD44	0.004	4.8
miR-16	0.001	3.4
miR-16-SNORD44	0.008	3.7
5S	0.0058	-23
5S-SNORD44	0.009	-21
SNORD44	0.708	-1.1
Cancer-free versus healthy		
U6	3.68 × 10^-5^	5.35
U6-SNORD44	0.00028	6.64
miR-16	0.0095	2.45
miR-16-SNORD44	0.015	3.04
5S	0.02	-11.0
5S-SNORD44	0.037	-9.0
SNORD44	0.39	-1.24
Metastasis versus cancer-free		
U6	0.59	-1.24
U6-SNORD44	0.53	-1.39
miR-16	0.38	1.37
miR-16-SNORD44	0.67	1.23
5S	0.51	-2.06
5S-SNORD44	0.46	-2.38
SNORD44	0.68	1.12

Comparisons were made between healthy subjects and patients with metastasis, healthy subjects and cancer-free patients, and patients with metastasis and those who were cancer-free (cohort 2). miR-16, microRNA 16; SNORD44, small nucleolar RNA 44.

The majority of patients in this cohort had ER/PR^+ ^breast cancer. We reanalyzed the above data by considering only ER^+^/PR^+ ^patients. Significantly elevated U6 or U6/SNORD44 ratio was still observed in cancer-free patients or patients with active metastasis compared with healthy subjects (Table 7).

**Table 7:** ER^+^/PR^+ ^subgroup-specific differences in serum U6, 5S, and other RNAs

Column ID	*P *value	Fold change
Metastasis versus healthy		
U6	0.0024	3.26
U6-SNORD44	0.003	4.18
miR-16	0.001	2.83
miR-16-SNORD44	0.002	3.65
5S	0.0027	-21
5S-SNORD44	0.007	-16
SNORD44	0.275	-1.3
Cancer-free versus healthy		
U6	3.09 × 10^-5^	5.4
U6-SNORD44	0.0002	6.35
miR-16	0.004	2.46
miR-16-SNORD44	0.01	2.9
5S	0.05	-7.2
5S-SNORD44	0.07	-6.16
SNORD44	0.51	-1.17
Metastasis versus cancer-free		
U6	0.16	-1.68
U6-SNORD44	0.37	-1.51
miR-16	0.64	1.14
miR-16-SNORD44	0.55	1.27
5S	0.268	-2.93
5S-SNORD44	0.326	-2.65
SNORD44	0.66	-1.11

Sera of patients with estrogen receptor/progesterone receptor-positive (ER/PR^+^) breast cancer were compared with those of healthy subjects (cohort 2). miR-16, microRNA 16; SNORD44, small nucleolar RNA 44.

To determine whether differences in U6 levels retain statistical significance in a larger cohort, we added sera from 60 healthy subjects and 53 patients who are cancer-free to the above cohort and measured U6 and SNORD44 levels. Characteristics of patients in this extended cohort are presented in Additional file 2. U6 levels in patients with breast cancer were significantly higher than those in healthy subjects (1.5-fold, *P *= 0.05) (Additional file 3). For unknown reasons, SNORD44 levels in the sera of approximately 20% of samples were undetectable. U6/SNORD44 ratio after excluding SNORD44^- ^samples (Additional file 2) remained significantly higher in patients with cancer compared with healthy subjects (2.3-fold, *P *= 0.03) (Additional file 3).

### Analysis of sera from a third cohort of patients

To confirm the results, we analyzed sera from 12 healthy subjects and 18 patients with active metastasis (cohort 3). U6 levels were elevated in sera of patients with active metastasis but did not reach statistical significance, possibly due to the smaller sample size of healthy subjects (Table 8). However, U6/SNORD44 ratio was significantly elevated in sera of patients with active metastasis compared with healthy subjects in this cohort. In this cohort, as with the second cohort, we observed significantly elevated miR-16 and miR-16/SNORD44 ratio in patients with active metastasis. However, 5S and 5S/SNORD44 ratio in cohorts 2 and 3 were incompatible with results from the first cohort. Overall, our analysis included sera from 115 healthy subjects, 107 clinically disease-free women, and 33 women with active metastasis. Taken together, these results reveal reproducible upregulation of serum U6 levels in women who experienced breast cancer, irrespective of disease activity at the time of serum collection.

**Table 8:** Differences in the levels of U6, 5S, and other RNAs between healthy subjects and cancer patients with active metastasis (cohort 3)

Column ID	*P *value	Fold change
U6	0.26	1.54
U6-SNORD44	0.043	2.28
miR-16	0.002	2.63
miR-16-SNORD44	0.0002	3.9
5S	0.037	-2.25
5S-SNORD44	0.25	-1.52
SNORD44	0.0124	-1.48

Patients in this cohort had active metastasis at the time of serum collection. miR-16, microRNA 16; SNORD44, small nucleolar RNA 44.

## Discussion

This study was designed to address two critical issues related to circulating miRNAs as a biomarker in breast cancer: the first concerned normalization control and the second was related to persistence of miRNA changes in patients who are clinically cancer-free. Although analysis of candidate miRNAs did not reveal major cancer-specific changes in serum profile, our results clearly showed elevated levels of U6 RNA, which is often used for normalization, in the sera of patients with breast cancer. With SNORD44 as a normalization control, we could demonstrate upregulation of U6 in the sera of both ER/PR^+ ^and ER/PR^- ^breast cancer patients who were in remission. This also indicates that the type of treatment has no effect on serum U6 levels as ER/PR^+ ^and ER/PR^- ^patients receive different therapies. Disease activity did not appear to influence the levels of serum U6 as sera from ER/PR^+ ^versus ER/PR^- ^or node-positive versus node-negative patients did not show a statistically significant difference in U6 levels. Sera of patients with active disease also showed elevated levels of U6. Further studies assessing U6 levels before and after treatment are required to test the temporal effects of treatment on serum U6 levels.

The above observations raise two important questions: one is related to the source of serum U6 RNA and the other is related to the mechanism(s) leading to altered U6 levels in serum. It is generally believed that tumor cells are the primary source of serum miRNAs [28]. Reduction of miR-92 in sera of patients with acute myeloid leukemia [19], reduction of 28 miRNAs in sera of patients with lung cancer [18], and our observation of elevated U6 RNA in sera of patients with metastasis-free breast cancer favor the possibility that cancer alters the release of miRNAs from distant organs or the immune system. The majority of plasma microvesicles are derived from leukocytes; therefore, cancer-induced alteration in leukocyte functions may potentially contribute to miRNA profile changes in the serum of patients with cancer [14].

Our observation of persistent change in U6 levels even after a patient is cancer-free is slightly surprising. It is possible that cancer-derived growth factors/cytokines, stress, host response to cancer, or carcinogens result in stable epigenetic changes in distant organs. In this context, it was recently reported that chronic stress induces epigenetic changes, which impact DNA methylation patterns and consequent effects on gene expression in both germline and somatic tissues [30]. Furthermore, neonatal experiences altering ERα levels in the adult mammary gland and consequent effects on mammary tumor incidence have been reported using animal models [31]. In addition, a recent study showed that individuals with a persistent asymptomatic hepatitis B virus (HBV) infection and patients with active HBV infection share a serum miRNA profile, which is distinct from a healthy individual [32]. Thus, a chronic infection/inflammatory condition may prompt certain organs to undergo permanent change in gene expression pattern. An alternative possibility, which may be provocative, is that upregulation of serum U6 levels is a preamble to cancer initiation or suggestive of a pre-cancerous state, similar to the creation of a niche for metastasis by the vascular endothelial growth factor receptor-1-positive (VEGFR-1^+^) hematopoietic bone marrow progenitor cells before the arrival of cancer cells [33].

RNAP-III transcribes both U6 and 5S RNA [34,35]. Aberrant RNAP-III-mediated transcription during cancer progression is just beginning to be recognized. RNAP-III upregulation is essential for cMyc-induced transformation [36]. The major signaling pathways activated in cancer, including Ras, Raf, PI3K, and AKT, enhance RNAP-III activity, whereas several tumor suppressors, including retinoblastoma, PTEN, p53, and BRCA1, decrease RNAP-III activity [35]. Inactivation of BRCA1 alone is sufficient to increase U6 levels in cancer cells [37]. Since we observed elevated U6 levels in the sera of patients who are cancer-free at the time of sample collection as well as in the sera of patients with metastasis, alteration in the RNAP-III transcription machinery may be one of the systemic changes that occur during cancer progression prior to diagnosis and treatment. Recent serum protein biomarker profiling studies have shown a 'chronic inflammatory state' in patients with breast cancer [38]. Whether such an inflammatory state alters serum U6 levels by modulating RNAP-III activity is not known. Unlike RNA polymerase II, RNAP-III has not been targeted for cancer therapy. It is not known whether serum U6 influences the course of the disease or whether blocking it will impact progression of the disease. It is also not known whether serum U6 is complexed with Argonaute2 or HDL and is delivered to heterotypic cells to modulate gene expression through alternative splicing [10,11]. We hope that our observations will prompt additional studies using inhibitors that can modulate (but not eliminate) the activity of RNAP-III to control cancer cell growth or the secondary effects of cancer or both.

## Conclusions

This is the first study to report aberrant levels of U6 small RNA in sera of patients with breast cancer. Since elevated U6 levels were observed in patients who are clinically cancer-free, serum U6 levels may serve as a 'surrogate marker' for permanent, cancer-induced systemic changes, irrespective of the disease course. Several challenges lie ahead; these are related to identifying the nature of these systemic changes as well as organs other than the primary site of cancer. Distinguishing cancer-associated deleterious systemic changes from insignificant changes is a huge challenge. Nonetheless, additional studies in this direction may help us to understand cancer as a systemic disease and potentially to develop treatment strategies targeting the 'organ component' and 'systemic component' of cancer.

## Abbreviations

CT: cycle threshold; ER: estrogen receptor; HBV: hepatitis B virus; HDL: high-density lipoprotein; miRNA: microRNA; PR: progesterone receptor; qPCR: quantitative polymerase chain reaction; RNAP-III: RNA polymerase III; SNORD44: small nucleolar RNA 44.

## Competing interests

The authors declare that they have no competing interests.

## Authors' contributions

HNA carried out sample preparation and qPCR. CPG and YL performed the statistical analysis. LAM, SB, and GWS participated in patient recruitment, data interpretation, and editing of the manuscript. HN designed the experiments, interpreted results, and wrote the manuscript. All authors read and approved the final manuscript.

## Supplementary Material

Additional file 1 **A detailed description of procedure used for serum collection**. Click here for file

Additional file 2 **Characteristics of healthy and breast cancer patients who are cancer free at the time of serum collection (extended cohort 2)**. Click here for file

Additional file 3 **Levels of U6 without (left) and with normalization to small nucleolar RNA 44 (SNORD44) (right) in the sera of healthy volunteers and breast cancer patients who are cancer free at the time of serum collection (extended cohort 2)**. U6 levels in the sera without (left) or after normalization (right) with SNORD44 for the extended cohort 2. Delta CT method (Ct average of U6 - Ct average of SNORD44) was used for this analysis. Lower the delta CT, higher the expression. Additional file 2 provides details of patient characteristics and number of samples used for this analysis. Click here for file
