# Supplementary material for: Persistent upregulation of U6:SNORD44 small RNA ratio in the serum of breast cancer patients
Source: Breast Cancer Res. 2011 Sep 13;13(5):R86. doi: 10.1186/bcr2943 (PMC3262198; doi:10.1186/bcr2943)
Supplement: Additional file 1 — A detailed description of procedure used for serum collection. [file bcr2943-S1.DOC]

**Standard operating procedure for serum collection.**

**Position for venipuncture**: sitting

**Materials:**

**Blood collection sets:** BD (Becton, Dickinson and Company) Vacutainer® Blood Collection Set, 21 gauge butterfly.

**Collection tube**: BD (Becton, Dickinson and Company) Vacutainer Venous Blood Collection Tubes: SST* Serum Separation Tubes Red/Gray top 8.5ml (Fisher cat. #0268396)

**Centrifuge:** Eppendorf 5702 or 5702R

**Cryostorage tubes:** Corning 2.0ml Cryogenic Vials. (Fisher cat. # 0337421)

**Repeater Pipet:** Eppendorf Repeater Plus Pipette (Fisher cat. # 21-380-9)

**Combitips:** (Fisher cat. #21-381-330)

**Labelling:** All tubes are to have bar code stickers placed on the tube prior to venipuncture. Bar code stickers will be generated during the process of registration of the volunteer donor.

**Order of the Blood Draw:** Blood collection tubes must be drawn in a specific order to avoid cross-contamination of additives between tubes. [4] The order of draw is 1) SST, 2) EDTA 9ml, and 3) EDTA 2ml. A total of three tubes of blood are drawn during the collection process.

**Temperature for collection and processing**: Blood samples to be separated into serum are drawn into an SST* and allowed to clot at room temperature.

**Processing:** Best Practice recommends that separation into serum and placement of serum into frozen storage should occur within 2 hours of the blood draw.

Blood is drawn into the Serum Separator tube and gently mixed by inverting the tube 5 times. Forty five minutes (±10 min.) after the blood has been drawn, the Serum Separator Tube is placed into the centrifuge and centrifuged at 2800rpm (1200g) for ten minutes at room temperature. A repeater pipet is used to aliquot 600ul of the serum into each of five cryogenic vials.

**Storage of Serum:**

Freeze-thaw is not optimal and therefore, serum should be aliquoted.

Serum aliquots are logged into cryoboxes and placed on dry ice for transport to the storage facility.

Serum is stored at -80^o^C.
