# Supplementary material for: Persistent upregulation of U6:SNORD44 small RNA ratio in the serum of breast cancer patients
Source: Breast Cancer Res. 2011 Sep 13;13(5):R86. doi: 10.1186/bcr2943 (PMC3262198; doi:10.1186/bcr2943)
Supplement: Additional file 2 — Characteristics of healthy and breast cancer patients who are cancer free at the time of serum collection (extended cohort 2). [file bcr2943-S2.DOC]

**Additional file 2:** **Characteristics of healthy volunteers and breast cancer patients who are cancer-free at the time of serum collection (extended cohort 2).** All patients in this group had measurable U6

|  | Healthy | All patients |
| --- | --- | --- |
| Number | 75 | 68 |
| Age  Mean:  Median:  Range | 49.74  39  30-84 | Diagnosis Analysis  44.9 51.33  44 51  24-72 31-76 |
| **Treatment**  Radiation:  Chemo:  Hormone: |  | 56  51  53 |
| **Characteristics of healthy volunteers and breast cancer patients in the extended cohort 2 with measurable U6 and SNORD44** | | |
|  | Healthy | All patients |
| Number | 60 | 55 |
| Age  Mean:  Median:  Range | 49.65  49  30-84 | Diagnosis Analysis  43.56 50.25  44 51  24-72 31-76 |
| **Treatment**  Radiation:  Chemo:  Hormone: |  | 44  38  41 |

SNORD44, small nucleolar RNA 44
