# Supplementary figures and images for: Persistent upregulation of U6:SNORD44 small RNA ratio in the serum of breast cancer patients
Source: Breast Cancer Res. 2011 Sep 13;13(5):R86. doi: 10.1186/bcr2943 (PMC3262198; doi:10.1186/bcr2943)

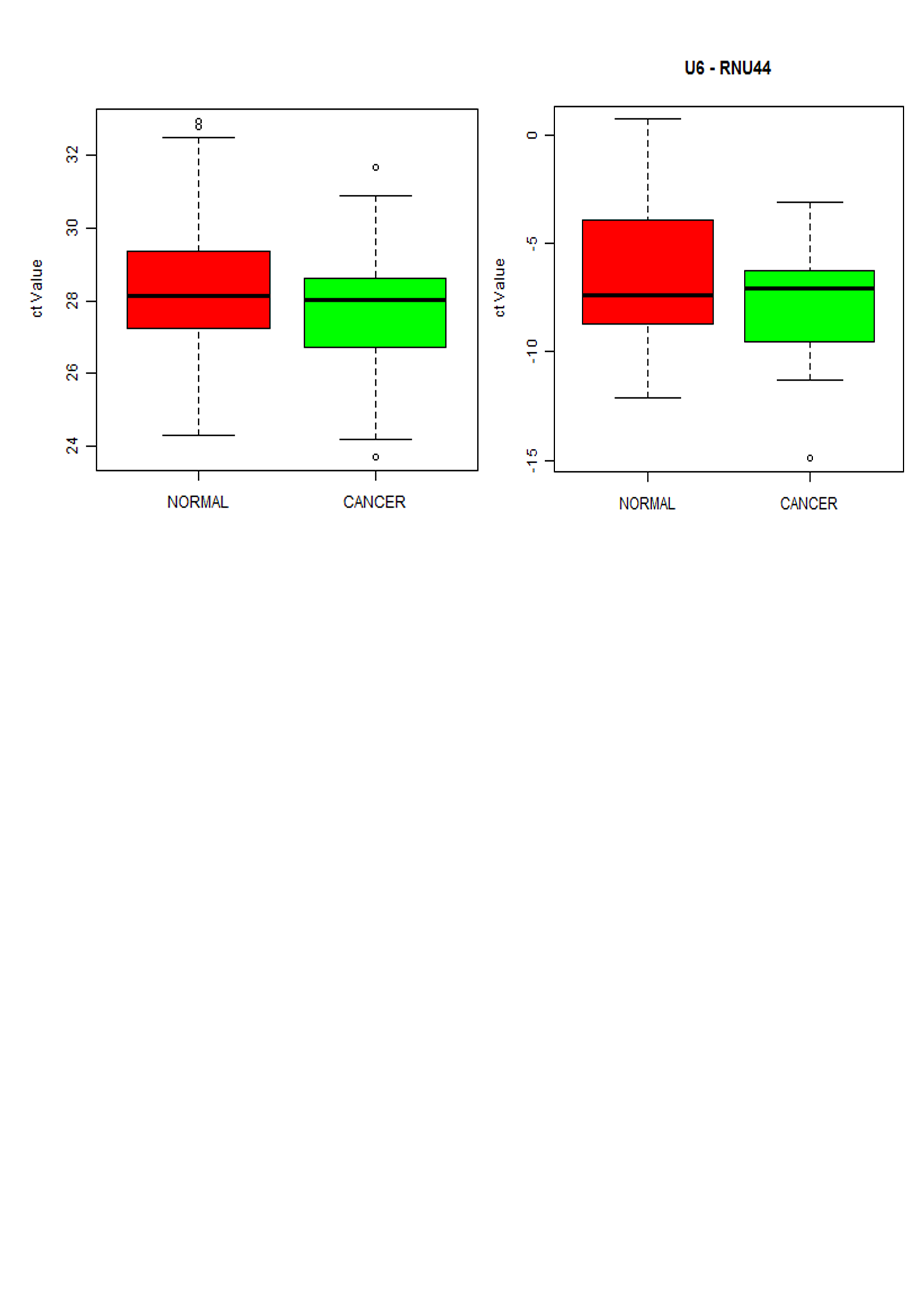

Supplement: Additional file 3 — Levels of U6 without (left) and with normalization to small nucleolar RNA 44 (SNORD44) (right) in the sera of healthy volunteers and breast cancer patients who are cancer free at the time of serum collection (extended cohort 2). U6 levels in the sera without (left) or after normalization (right) with SNORD44 for the extended cohort 2. Delta CT method (Ct average of U6 - Ct average of SNORD44) was used for this analysis. Lower the delta CT, higher the expression. Additional file 2 provides details of patient characteristics and number of samples used for this analysis. [file bcr2943-S3.TIFF]
